# The frequency of CD127^low ^expressing CD4^+^CD25^high ^T regulatory cells is inversely correlated with human T lymphotrophic virus type-1 (HTLV-1) proviral load in HTLV-1-infection and HTLV-1-associated myelopathy/tropical spastic paraparesis

**DOI:** 10.1186/1471-2172-9-41

**Published:** 2008-07-29

**Authors:** Jakob Michaëlsson, Hugo Marcelo R Barbosa, Kimberley A Jordan, Joan M Chapman, Milena KC Brunialti, Walter Kleine Neto, Youko Nukui, Ester C Sabino, Marco Antonio Chieia, Acary Souza Bulle Oliveira, Douglas F Nixon, Esper G Kallas

**Affiliations:** 1 Division of Experimental Medicine, Department of Medicine, University of California, San Francisco, San Francisco, CA, USA; 2 Infectious Diseases Division, Federal University of São Paulo, São Paulo, Brazil; 3 São Paulo Blood Bank, São Paulo, Brazil; 4 Clinical Immunology and Allergy Division, University of São Paulo, Brazil; 5 Center for Infectious Medicine, Department of Medicine, Karolinska Institutet, Stockholm, Sweden

## Abstract

**Background:**

CD4^+^CD25^high ^regulatory T (T_Reg_) cells modulate antigen-specific T cell responses, and can suppress anti-viral immunity. In HTLV-1 infection, a selective decrease in the function of T_Reg _cell mediated HTLV-1-tax inhibition of FOXP3 expression has been described. The purpose of this study was to assess the frequency and phenotype of T_Reg _cells in HTLV-1 asymptomatic carriers and in HTLV-1-associated neurological disease (HAM/TSP) patients, and to correlate with measures of T cell activation.

**Results:**

We were able to confirm that HTLV-I drives activation, spontaneous IFNγ production, and proliferation of CD4+ T cells. We also observed a significantly lower proportion of CTLA-4^+ ^T_Reg _cells (CD4^+^CD25^high ^T cells) in subjects with HAM/TSP patients compared to healthy controls. Ki-67 expression was negatively correlated to the frequency of CTLA-4^+ ^T_Reg _cells in HAM/TSP only, although Ki-67 expression was inversely correlated with the percentage of CD127^low ^T_Reg _cells in healthy control subjects. Finally, the proportion of CD127^low ^T_Reg _cells correlated inversely with HTLV-1 proviral load.

**Conclusion:**

Taken together, the results suggest that T_Reg _cells may be subverted in HAM/TSP patients, which could explain the marked cellular activation, spontaneous cytokine production, and proliferation of CD4^+ ^T cells, in particular those expressing the CD25^high^CD127^low ^phenotype. T_Reg _cells represent a potential target for therapeutic intervention for patients with HTLV-1-related neurological diseases.

## Background

Between 10 and 20 million people are infected with HTLV-1 worldwide [1]. Although most subjects are clinically asymptomatic during their lifetime, a proportion (5 to 10%) develop adult T cell leukemia/lymphoma (ATLL) or HTLV-1 associated myelopathy/tropical spastic paraparesis (HAM/TSP) [2]. Epidemiological surveys have identified regions in the world where prevalence rates are considerably higher, including Japan, the Caribbean, South America, Africa, Melanesia and the Middle East [1,3]. It has been estimated that the prevalence of HTLV-1 infection in South America ranges from 2 to 5% [4], with an estimated 1–2 million infected people in Brazil [5]. The prevalence in blood donors ranges from 0.17 to 1.8% in different areas of the country [6,7], with a 0.3% seroprevalence in the city of Sao Paulo blood donors [8].

HTVL-1 is a retrovirus encoding the group specific antigen (*gag*), protease (*pro*), polymerase (*pol*), and envelope (*env*) genes. Six proteins are encoded by the pX region of the genome, including the Tax protein, which is critical to viral replication and induction of cellular activation and transformation, increasing the expression and production of cytokines and receptors involved in T cell growth and transformation, such as IL-15 [9,10] and IL-2 [11-13]. Tax also has the ability of interfering in the expression of several transcription factors and proto-oncogenes, as well as in the nucleic acid repair and apoptosis [14-17]. These effects combined seem to play a key role in the potential of HTLV-1 to induce cellular transformation and, consequently, trigger the development of ATLL.

It has been previously demonstrated that HTLV-1 proviral load is one of the key factors in the pathogenesis of HAM/TSP [18,19], although host genetic factors are also independently associated with the development of the diseases, e.g. certain HLA [20,21] and non-HLA [22,23] genes. These invoke the hypothesis that both viral and genetic host factors are implicated in the pathogenesis of HAM/TSP.

The CD8^+ ^T cell response to HTLV-1 can be readily detected [24-31], commonly directed against the HTLV-1-tax protein. The contribution of the CD8^+ ^T cell response might be particularly important for viral control in HTLV-1 infection, since infected lymphocytes produce virtually no cell-free infectious HTLV-1 particles. However, it is noteworthy that the magnitude of the HTLV-1-specific T cell response is associated with higher proviral loads, highlighting the fact that T cells frequencies are determined by proviral load, as well as being a determinant of proviral load. CD4^+ ^T cells are the main target for HTLV-1 infection, which induces CD4^+ ^T cell activation, including proliferation and IFNγ production. The HTLV-1-specific CD4^+ ^T cell response is directed mainly against Env, the HTLV-1 envelope surface [32].

T_Reg _cells are crucial for the control of autoimmune disease and maintenance of peripheral T cell tolerance (reviewed in Sakagushi et al. [33]). In addition, they can suppress pathogen-specific T cell responses, including response to viruses [34-37]. The mechanisms whereby T_Reg _cells suppress T cell responses are not yet fully understood, but are likely to include both soluble factors, e.g. IL-10 and TGF-β, as well as cell-cell contact dependent mechanisms, e.g. through CTLA-4. CTLA-4 (CD152) is expressed by a large fraction of CD4^+^CD25^+ ^T cells, and by a majority of CD4^+^CD25^high ^T cells. CTLA-4 has also been shown to be one of mediators of T_Reg _function [38,39], and is considered a marker for T_Reg _cells. In addition, it was recently demonstrated that T_Reg _cells are characterized by low levels of the IL-7Rα (CD127^low^) [40-42], which together with CD25 help to distinguish T_Reg _cells from activated normal CD4^+ ^T cells in healthy individuals. FOXP3 is a key regulator of T_Reg _cell function, but is not exclusive to T_Reg _cells; it has been identified in human nonregulatory activated CD4^+^FoxP3^+ ^T cells. Humans with mutations in FOXP3 present with a syndrome characterized by severe autoimmune and inflammatory disorders often early in life, denominated IPEX [33]. Interestingly, it was recently shown that HTLV-1 tax can downregulate Foxp3 expression [43,44].

We hypothesized that HTLV-1 compromises T_Reg _cell function, resulting in higher T cell activation, which contributes to HAM/TSP development. We found a significantly higher frequency of CD4^+^Ki-67^+ ^T cells and a lower proportion of CTLA-4^+ ^T_Reg _cells in subjects with HAM/TSP, compared to healthy controls. Moreover, we found an inverse correlation between HTLV-1 proviral load and frequency of CD127^low^/CTLA-4^+^T_Reg _cells. Our data suggest a role for T_Reg _cells in the pathogenesis of HAM/TSP, and reveal a potential new therapeutic target for patients with HAM/TSP.

## Results

### Study subjects

Blood samples were collected at the Federal University of Sao Paulo outpatient clinics, after informed consent. PBMC were isolated by Ficoll-Paque PLUS density gradient centrifugation and cryopreserved. The demographics of the study subjects are shown in Table 1, including gender, age, proviral load, CD3, CD4 and CD8 absolute T cell counts. No statistically significant differences were observed in gender and age distribution among groups.

**Table 1:** Characteristics of study subjects.

**Sample**	**Group**	**Gender**	**Age (years)**	**Proviral Load (copies/1,000 cells)**	**CD4+ T cells/μl**	**CD8+ T cells/μl**	**CD3+ T cells/μl**
101	Control	Male	34	NA	690	389	1220
102	Control	Male	-	NA	1116	525	1649
103	Control	Male	-	NA	-	-	-
104	Control	Male	-	NA	-	-	-
105	Control	Female	31	NA	-	-	-
106	Control	Female	28	NA	-	-	-
107	Control	Male	49	NA	917	233	1235
201	HTLV+	Female	23	60	1321	619	1970
202	HTLV+	Female	49	183	710	700	1402
203	HTLV+	Male	40	-	548	351	989
204	HTLV+	Female	33	314	749	494	1248
205	HTLV+	Female	66	120	705	227	934
206	HTLV+	Female	31	105	738	482	1341
209	HTLV+	Female	33	11	1274	622	1909
212	HTLV+	Male	31	160	904	527	1500
213	HTLV+	Female	37	7	1320	496	1831
214	HTLV+	Male	62	19	535	251	782
301	HAM/TSP	Female	47	723	735	354	1092
302	HAM/TSP	Female	54	-	1012	242	242
303	HAM/TSP	Male	30	-	-	-	-
304	HAM/TSP	Female	56	Undetectable	727	356	1076
305	HAM/TSP	Female	50	75	563	327	972
306	HAM/TSP	Female	26	151	881	334	1272
307	HAM/TSP	Female	43	Undetectable	899	500	1472
308	HAM/TSP	Female	55	433	1154	478	1694
309	HAM/TSP	Female	NA	175	614	165	520
310	HAM/TSP	Female	34	565	734	286	1039

### CD4+ T cell activation and IFNγ production in healthy donors, HTLV-1 seropositive asymptomatics, and HAM/TSP patients

**Figure 1:**
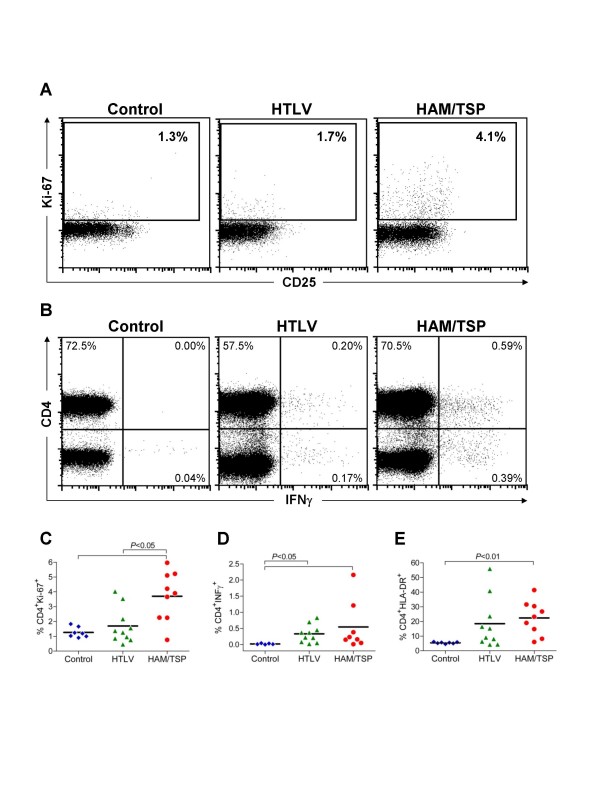
**Expression of Ki-67, HLA-DR and spontaneous production of IFNγ by CD4^+ ^T cells from Control, HTLV and HAM/TSP subjects.** (A) Representative dot plots showing Ki-67 expression in CD4^+ ^T cells from a Control, a HTLV, and a HAM/TSP subject. (B) Representative dot plots showing spontaneous IFNγ production by CD4^+ ^T cells. (C) Percent CD4^+^Ki-67^+ ^T cells; and (D) percent CD4^+^IFNγ^+ ^T cells; or (E) percent CD4^+^HLA-DR^+ ^T cells from Control, HTLV, or HAM/TSP. The line shown represents the mean.

We initially investigated the expression of Ki-67, HLA-DR and CD38 on CD4^+ ^T cells in PBMC from healthy donors (Control), HTLV-1 infected patients who were clinically asymptomatic (HTLV), or had associated neurological disease (HAM/TSP). HAM/TSP had significantly higher frequencies of CD4^+^Ki-67^+ ^T cells compared to HTLV or Control subjects (Fig. 1A, 1C). In addition, HAM/TSP patients had an increase in the frequency of CD4^+ ^HLA-DR^+ ^T cells compared to Controls (Fig. 1E), whereas no statistically significant difference in frequency of CD4^+^CD38^+ ^T cells was noted (data not shown). Furthermore, CD4^+ ^T cells from both HTLV and HAM/TSP groups had an increase in the spontaneous expression of IFNγ (Fig. 1B, 1D).

### Decreased frequency of T_Reg _cells in HAM/TSP patients

**Figure 2:**
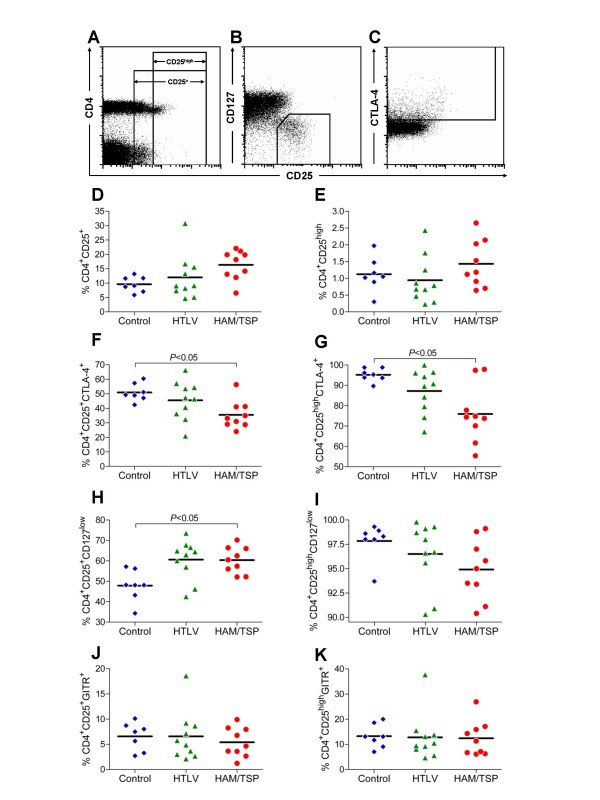
**Immunophenotypes of CD25^+ ^or CD25^high ^CD4^+ ^T cells in Control, HTLV, or HAM/TSP subjects.** Dot plots depict the gating strategies for CD25^+ ^and CD25^high ^cells (A); CD127^low ^(B); and CTLA-4 expression (C) are shown. The gates for CD25^+ ^and CD4^+^CD25^high ^T cells were set based on the expression level of CD25 on CD4^- ^T cells, where the pattern of CD25 expression is more distinct. Accordingly, CD4^+^CD25^high ^T cells were defined as those expressing CD25 at a level higher than the bulk CD4^-^CD25^+ ^population. For D-K, individual results are represented in symbols, and the line represents the mean. (D) % CD4^+^CD25^+^T cells; (E) % CD4^+^CD25^high ^T cells; (F) % CD4^+^CD25^+^CTLA-4^+ ^T cells; (G) % of CD4^+^CD25^high^CTLA-4^+ ^T cells; (H) % CD4^+^CD25^+^CD127^low ^T cells; (I) % CD4^+^CD25^high^CD127^low ^T cells; (J) % CD4^+^CD25^high^GITR^+ ^T cells; and (K) % CD4^+^CD25^high^GITR^+ ^T cells.

In order to assess the frequency of T_Reg _cells in HTLV-1 infected subjects, we measured the expression of CD25, CTLA-4, CD127 and GITR on CD4^+ ^T cells by flow cytometry. Gating strategies are shown in Fig. 2A–C. The frequency of CD4^+ ^T cells expressing CD25 was very similar between the groups (Fig. 2D). As CD25 is upregulated on activated CD4^+ ^T cells, and thus is not a specific marker for T_Reg _cells, we sought to determine the frequency of T_Reg _cells using more specific phenotypes. First, we analyzed the frequency of CD4^+^CD25^high ^T cells, known to be composed mainly of T_Reg _cells [45], and no significant difference in the frequency of T_Reg _cells could be found between controls and HTLV-1 subjects (Fig. 2E). We determined the intracellular expression of CTLA-4 in CD4^+^CD25^+^, and CD4^+^CD25^high ^T cells and observed a decrease in frequency of CTLA-4^+ ^from HAM/TSP patients (Fig. 2F and 2G). Next, we assessed the expression of CD127. HAM/TSP patients had a statistically significant increase in the frequency of CD4^+^CD25^+^CD127^low ^T cells compared to Controls (Fig. 2H). In contrast, there was a slight decrease in frequency of CD4^+^CD25^high^CD127^low ^T cells in HTLV-1 infected groups, although this did not reach statistical significance (Fig. 2I). There were no differences in percent expression of GITR between the groups (Fig. 2J, 2K).

### Decreased CTLA-4+ T_Reg _cells correlate with increased CD4+ T cell proliferation in HAM/TSP patients

**Figure 3:**
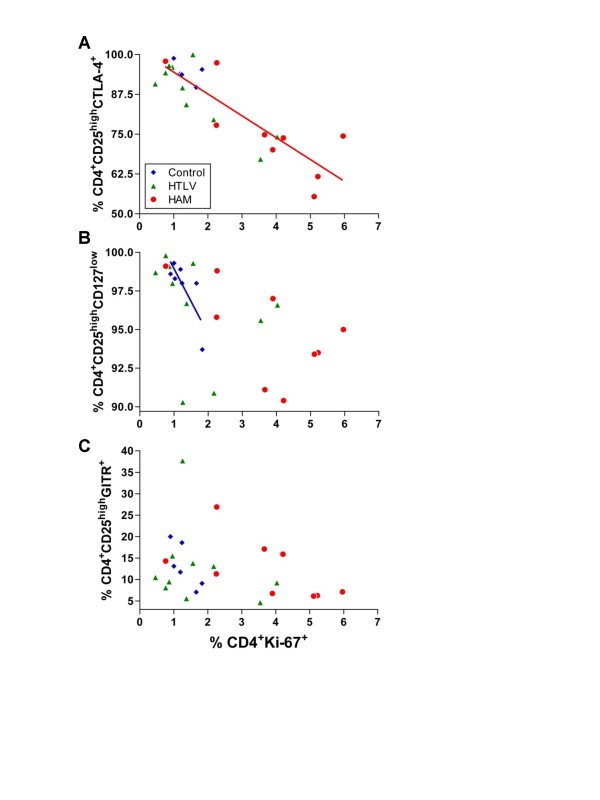
**Correlation between different immunophenotypes of T_reg _with expression of Ki-67.** Frequency of CTLA-4^+^, CD127^low^, or GITR^+ ^T_reg _were correlated with proportion of CD4^+^Ki67^+ ^T cells in Control (Blue diamond), HTLV (Green triangle) and HAM/TSP (Red circle). A. There was a negative correlation between CTLA-4^+ ^T_reg _with percentage of CD4^+^Ki67^+ ^T cells in HAM/TSP only (r = -0.7833, *p *= 0.017). B. CD127^low ^T_reg _were inversely correlated with proportion of CD4^+^Ki67^+ ^T cells in Control group (r = -0.8108, *p *= 0.034). C. There was no association between GITR^+ ^T_reg _with percentage of CD4^+^Ki67^+ ^T cells in any group.

Our initial analysis indicated that the frequency of CD4^+^CD25^+^CD127^low ^T cells was higher in HAM/TSP patients. However, there were a lower percentage of CTLA-4^+ ^T_Reg _cells, indicating that HAM/TSP subjects might have T_Reg _cells with a dysfunctional phenotype. Moreover, we showed that HAM/TSP patients had an increased proliferation and T cell activation, as evidenced by higher frequencies of HLA-DR, Ki-67 and INFγ-expressing CD4^+ ^T cells. To test whether the higher frequency of T_Reg _cells was associated with lower levels of activation or proliferation of CD4^+ ^T cells, we compared the frequency of CTLA-4^+^, CD127^low ^or GITR^+ ^T_Reg _cells with the percentage of CD4^+^Ki-67^+ ^T cells. The frequency of CTLA-4^+ ^T_Reg _was negatively correlated with the frequency of CD4^+^Ki-67^+ ^T cells in HAM/TSP patients only (Fig. 3A). In addition, there was a negative correlation between the frequency of CD127^low ^T_Reg _cells and the percentage of CD4^+^Ki-67^+ ^T cells in controls, whereas no such correlation was found in HTLV-1 infected subjects (Fig. 3B). There was no association between GITR^+ ^T_Reg _cells with the percentage of CD4^+^Ki-67^+ ^T cells in any group (Fig. 3C).

### The frequency of CD4^+^CD25^+^CTLA-4^+ ^and CD127^low ^T_Reg _cells was negatively correlated to HTLV-1 proviral load

**Figure 4:**
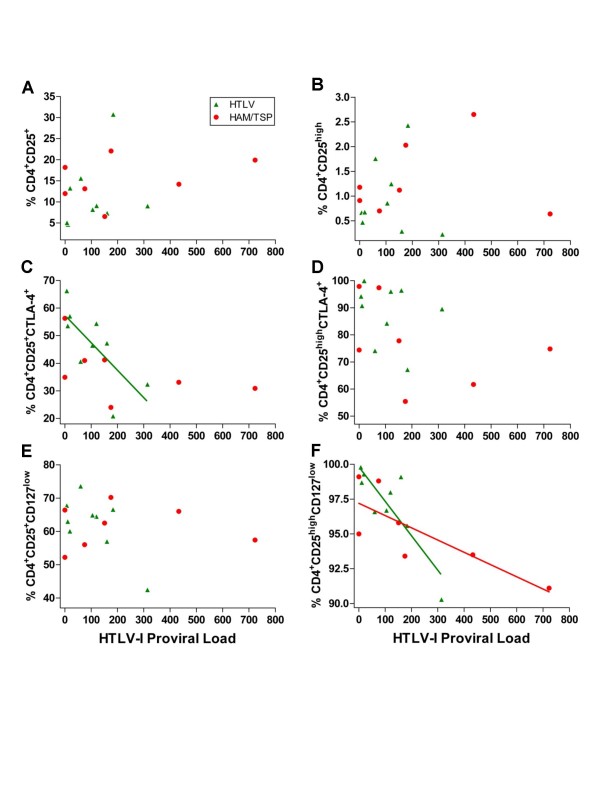
**Correlation between HTLV-1 proviral load of HTLV (Green triangle), and HAM/TSP (Red circle) with expression of CD127 or CTLA-4 in CD25^+ ^or CD25^high ^CD4^+ ^T cells subsets.** HTLV-I proviral load is expressed in copies per 1,000 cells. There was no correlation with CD4^+^CD25^+ ^T cells (A). No correlation with CD4^+^CD25^high ^T cells (B). Inverse correlation with the percentage of CD4^+^CD25^+^CTLA-4^+ ^T cells in HTLV only; r = -0.7500, *p *= 0.0255 (C). No correlation with CD4^+^CD25^high^CTLA-4^+ ^T cells (D). Lack of correlation with the percentage of CD4^+^CD25^+^CD127^low ^T cells (E). (F) Negative correlation with CD4^+^CD25^high^CD127^low ^T cells in HTLV (r = -0.7333, *p *= 0.03) and HAM/TSP (r = -0.8108, *p *= 0.034).

Finally, we wanted to determine whether the frequency of T_Reg _cells was related to HTLV-1 proviral load. High CD4^+ ^T cells activation and elevated HTLV-1 proviral load are observed in HAM/TSP. We hypothesized that this phenomenon would be related to a lower proportion of T_Reg _cells. We quantified HTLV-1 proviral load by real-time PCR and correlated it with the frequency of the different CD25-expressing CD4^+ ^T cell subsets. There was no correlation between the frequency of CD4^+^CD25^+ ^or CD4^+^CD25^high ^T cells with proviral load (Fig. 4A and 4B). In contrast, there was a negative correlation between the frequency of CD4^+^CD25^+^CTLA-4^+ ^T cells and proviral load in HTLV only (Fig. 4C). Moreover, there was an inverse correlation between CD127^low ^T_Reg _cells and HTLV-1 proviral load (Fig. 4F).

## Discussion

Regulatory T cells are important for the maintenance of peripheral T cell tolerance to self antigens, and can also suppress T cell responses to tumors, parasites, viruses and bacteria. In this study we addressed the relationship between T_Reg _cells, T cell activation, and HTLV-1 proviral load. Infection with HTLV-1 was associated with higher spontaneous IFNγ release by CD4^+ ^T cells, but only in HAM/TSP there was a marked increase in T cell proliferation.

The HTLV-1 derived tax protein can downregulate expression of the FOXP3, which presence is associated with T_Reg _cell function [43,44]. Increased expression of tax can be expected in patients with HAM/TSP, who have higher proviral loads compared to asymptomatic carries. We observed a higher proportion of CD4^+ ^IFNγ^+ ^T cells in HTLV-1 infected subjects, which could also be indicative of a decreased T_Reg _cell fraction. Interestingly, only the HAM/TSP patients presented with a higher cell proliferation, as measured by Ki-67 staining, which correlated markedly with HTLV-1 proviral load (data not shown). These observations suggest that HTLV-1 directly affects T_Reg _cell number, and as proviral load increases, not only is the control of IFNγ lost, but controls on cell proliferation as well. Our data, together with the recent findings that HTLV-1 tax downregulates FOXP3 expression, indicate that T_Reg _cell dysfunction can be a direct consequence of HTLV-1 infection.

In order to better understand the role of T_Reg _cells in HTLV-I infection and disease, we used CTLA-4 and CD127 staining in CD4^+^CD25^high ^cells as markers for T_Reg _subsets. CD127 and CTLA-4 have been described as useful markers for T_Reg_, and facilitate the identification of T_Reg _cells, even without staining for FOXP3 [40]. In this study, we found that an increased frequency of CD127^low^CD4^+^CD25^+ ^T_Reg _in controls correlated negatively with CD4^+ ^T cell proliferation (Ki-67), indicating that these cells indeed have a regulatory immunophenotype. In contrast, increased frequency of these cells correlated with increased CD4^+ ^T cell proliferation in HTLV-1 infected individuals, suggesting that these cells are not regulatory T cells in these individuals. In addiction, the elevated frequency of CTLA-4^+ ^T_Reg _cells was negatively correlated to CD4^+ ^T cell proliferation only in HAM/TSP patients, which suggest that it is a better immunophenotype of T_Reg _cells in HAM/TSP patients, but more studies are necessary to confirm this.

We could detect a negative association between the frequency of CTLA-4^+ ^or CD127^low ^T_Reg _cells and proviral load, extending recent findings of an association between FOXP3 expression and HTLV-1 infection [44]. We speculate that therapeutic manipulation of regulatory T cells could positively impact disease pathogenesis. Two mechanisms might be involved, the first by suppressing the exuberant anti-HTLV-1 CD8^+ ^T cell mediated immune response, and the second by suppression of CD4^+ ^T cell proliferation, which can result in lower proviral load. However, stimulating an expansion of T_Reg _cells could also provide additional targets for HTLV-1 replication, so such studies should proceed with great caution.

In this study, there are some limitations. The study was cross sectional, and with a limited number of patients in each group. We hope that future longitudinal studies can assess changes in T_Reg _cells over time in HTLV-1 infected patients. We, and others, working in the regulatory T cells field, are limited by the lack of definitive phenotypic markers of T_Reg_, and CD4^+^CD25^+/high ^remains the standard identifiers. In this study we have added other markers, but at the time the study was conducted, the FOXP3 antibody, commonly used to detect a T_Reg _cell population was not commercially available. However, this may have been a fortuitous event, as recent reports suggest that FOXP3 is also expressed on non regulatory T cells in humans [46,47]. As we did not have access to tissue samples from these subjects, we cannot exclude redistribution of cells out of the peripheral blood into tissues, and the study of regulatory T cells at secondary lymphoid sites and within CSF will be of interest for a future study of HTLV-1 associated disease.

In conclusion, our data suggest a role of T_Reg _cells in the pathogenesis of HAM/TSP. Further studies should help delineate the ability of expanded T_Reg _cells to affect T cell proliferation in HTLV-1 patients and the potential development of therapeutic modulation of regulatory T cells in HTLV-1 patients.

## Conclusion

In this study, we showed that HTLV-I drives activation, spontaneous IFNγ production, and proliferation of CD4+ T cells. HAM/TSP patients have a decreased frequency of T_Reg _cells in peripheral blood, compared to healthy subjects, markedly in the CD4^+^CD25^high^CTLA^+ ^phenotype. The proportion of CD127^low ^T_Reg _cells correlated inversely with HTLV-1 proviral load. These results suggest that T_Reg _cells may be subverted in HAM/TSP patients, and contributes to the identification of novel therapeutic targets for patients with HTLV-1-related disease.

## Methods

### Study subjects

Three groups of volunteers were enrolled. The first consisted of seven HTLV-1-negative control volunteers; the second consisted of ten HTLV-1 seropositive volunteers without clinical and laboratory evidence of HTLV-1-associated disease, and the last group was composed of nine patients with the diagnosis of HTLV-1 associated myelopathy/tropical spastic paraparesis (HAM/TSP). After approval by the Institutional Review Board, written informed consent was obtained from all the participants according to the guidelines of Brazilian Ministry of Health. Samples were collected in EDTA-treated vacuum tubes, and PBMC were frozen into liquid nitrogen after separation using a ficoll gradient.

### DNA extraction and determination of HTLV-1 proviral load

HTLV-1 proviral DNA was extracted from PBMCs using a commercial kit (Qiagen GmbH, Hilden Germany) following the manufacturer's instructions. The extracted DNA was used as a template to amplify a fragment of 158 bp from the viral tax region using previously published primers [48]. The SYBR green real-time PCR assay was carried out in 25 μl PCR mixture containing 10× Tris (pH 8.3; Invitrogen, Brazil), 1.5 mM MgCl_2_, 0.2 μM of each primer, 0.2 mM of each dNTPs, SYBR Green (18.75 Units/r × n; Cambrex Bio Science, Rockland, ME) and 1 unit of platinum Taq polymerase (Invitrogen, Brazil). The amplification was performed in the Bio-Rad iCycler iQ system using an initial denaturation step at 95°C for 2 minutes, followed by 50 cycles of 95°C for 30 seconds, 57°C for 30 seconds and 72°C for 30 seconds. The human housekeeping β globin gene primers GH20 and PC04 [49] were used as an internal control calibrator. For each run, standard curves for the value of HTLV-1 tax were generated from MT-2 cells of log_10 _dilutions (from 10^5 ^to 10^0 ^copy). The threshold cycle for each clinical sample was calculated by defining the point at which the fluorescence exceeded a threshold limit. Each sample was assayed in duplicate and the mean of the two values was considered as the copy number of the sample. The amount of HTLV-1 proviral load was calculated as follows: copy number of HTLV-1 (tax) per 1,000 cells = (copy number of HTLV-1 tax)/(copy number of β globin/2) × 1000 cells. The method could detect 1 copy per 10^3 ^PBMCs cells.

### Flow cytometry

PBMCs were thawed and stained with directly conjugated antibodies. Three different panels of antibodies were used to evaluate the expression of proteins associated with T_Reg _cells and T cell activation. All antibodies were from BD Biosciences, unless otherwise noted. All panels contained PerCP-conjugated anti-CD4 and allophycocyanin-conjugated anti-CD25, and in addition contained (1) FITC-conjugated anti-GITR and PE-conjugated anti-CD127 (Beckman Coulter, Miami, FL), (2) FITC-conjugated anti-CD45RA and PE-conjugated anti-HLA-DR and (3) FITC-conjugated anti-Ki-67 and PE-conjugated anti-CD152 (CTLA-4). Cells stained with PerCP-conjugated anti-CD4 alone and allophycocyanin-conjugated CD25 alone were used to establish positive gates for FITC- and PE-conjugated antibodies. For panel 1 and 2, cells were stained with all antibodies in PBS supplemented with 0.5% bovine serum albumin (BSA) and 2 mM EDTA (FACS buffer), followed by two washes in FACS buffer and fixation in 1% paraformaldehyde (PFA). For panel 3, cells were first stained with PerCP-conjugated anti-CD4 and allophycocyanin-conjugated anti-CD25, followed by two washes in FACS buffer and fixation in 1% PFA. The cells were subsequently washed twice with PBS containing 0.1% saponin (perm buffer), prior to staining with PE-conjugated anti-CD152 and FITC-conjugated anti-Ki-67 diluted in perm buffer. All samples were analyzed on a FACSCalibur flow cytometer (Becton Dickinson) equipped with a 488 nm argon and a 633 nm red-diode lasers for four color detection. Acquisition and analyses were performed using CellQuest software (Becton Dickinson). Fluorescence voltages and compensation values were determined using unstained cells and cells single-stained with each of the fluorochrome-conjugated antibodies, respectively. The gating strategy used was to gate on lymphocytes using a forward scatter versus side scatter gate, followed by gating on CD4^+ ^cells. The gate for CD4^+^CD25^+ ^cells was set using cells cells stained with the PerCP-conjugated anti-CD4 antibody alone. Positive gates for the FITC- and PE-conjugated antibodies were set using cells stained with only PerCP-conjugated anti-CD4 and APC-conjugated anti-CD25 antibodies.

### Cytokine flow cytometry

PBMCs were thawed and cultured for 24 hours in 96-well U-bottom plates at a concentration of 4 × 10^5 ^cells/well. Brefeldin A (BFA) was added at a concentration of 5 μg/ml for the last 5 hours of the culture. After culture, cells were harvested, stained with PE-conjugated anti-CD4, fixed in 4% PFA for 20 min, prior to being washed twice with perm buffer. The cells were subsequently stained with PerCP-conjugated anti-CD3 and allophycocyanin-conjugated anti-IFNγ, washed twice in perm buffer and resuspended in FACS buffer, prior to being analyzed on a FACSCalibur. All antibodies were from BD Biosciences.

### Statistical analyses

Data sets were compiled and analyzed in Statistica, release 6.0 (Statsoft, Tulsa, OK) and Prism, release 4.0 (GraphPad Software, San Diego, CA). Groups comparisons were performed using non-parametric Kruskal Wallis ANOVA by ranks test; associations between variables were evaluated by Spearman rank order correlation's test. Critical *p *values were considered statistically significant if below 0.05.

## Competing interests

The authors declare that they have no competing interests.

## Authors' contributions

JM planed and conducted the experiments, performed the statistical analyses, and wrote the manuscript; HMRB wrote the original protocol, collected the samples, prepared the database, helped in the experiments, performed statistical analyses, and wrote the manuscript; KAJ conducted the experiments and helped in the flow cytometry analyses; JMC conducted the experiments and helped in the flow cytometry analyses; MKCB conducted the experiments and helped in the flow cytometry analyses; WKN performed the HTLV-I viral load; YN supported the protocol design and the conduction of HTLV-I viral load; ECS performed the HTLV-I serology, helped in the project design, the molecular biology experiments, and writing the manuscript; MAC selected subjects for the cohort and supervised the blood collection of samples from HAM/TSP patients; ASBO selected subjects for the cohort and supervised the blood collection of samples from HAM/TSP patients; DFN participated in the experimental design, obtained funds for the project, discussed the results, and wrote the manuscript; EGK wrote the original protocol, participated in the experimental design, obtained funds for the project, discussed the results, and wrote the manuscript.
